# Ammonolysis‐Driven Exsolution of Ru Nanoparticle Embedded in Conductive Metal Nitride Matrix to Boost Electrocatalyst Activity

**DOI:** 10.1002/advs.202309819

**Published:** 2024-04-06

**Authors:** So Yeon Yun, Sangseob Lee, Xiaoyan Jin, Aloysius Soon, Seong‐Ju Hwang

**Affiliations:** ^1^ Department of Materials Science and Engineering College of Engineering Yonsei University Seoul 03722 Republic of Korea; ^2^ Center for Artificial Synesthesia Materials Discovery Department of Materials Science and Engineering Yonsei University Seoul 03722 Republic of Korea; ^3^ Department of Applied Chemistry University of Seoul Seoul 02504 Republic of Korea

**Keywords:** electrocatalyst, holey nanotube, metal nanoparticle, metal nitride matrix, nitridation‐driven exsolution

## Abstract

Exsolution is an effective method for synthesizing robust nanostructured metal‐based functional materials. However, no studies have investigated the exsolution of metal nanoparticles into metal nitride substrates. In this study, a versatile nitridation‐driven exsolution method is developed for embedding catalytically active metal nanoparticles in conductive metal nitride substrates via the ammonolysis of multimetallic oxides. Using this approach, Ti_1−x_Ru_x_O_2_ nanowires are phase‐transformed into holey TiN nanotubes embedded with exsolved Ru nanoparticles. These Ru‐exsolved holey TiN nanotubes exhibit outstanding electrocatalytic activity for the hydrogen evolution reaction with excellent durability, which is significantly higher than that of Ru‐deposited TiN nanotubes. The enhanced stability of the Ru‐exsolved TiN nanotubes can be attributed to the Ru nanoparticles embedded in the robust metal nitride matrix and the formation of interfacial Ti^3+^─N─Ru^4+^ bonds. Density functional theory calculations reveal that the exsolved Ru nanoparticles have a lower *d*‐band center position and optimized hydrogen affinity than deposited Ru nanoparticles, indicating the superior electrocatalyst performance of the former. In situ Raman spectroscopic analysis reveals that the electron transfer from TiN to Ru nanoparticles is enhanced during the electrocatalytic process. The proposed approach opens a new avenue for stabilizing diverse metal nanostructures in many conductive matrices like metal phosphides and chalcogenides.

## Graphical abstract

Here, the development of a versatile nitridation−exsolution route to robust high‐performance electrocatalyst via the ammonolysis‐driven embedding of Ru metal nanoparticles in holey TiN nanotubes is reported. The exsolved Ru─TiN nanohybrid with strong interfacial interaction shows outstanding stable electrocatalytic activity for the hydrogen evolution reaction, which is attributable to the improvement of charge/mass transport and optimization of electronic structure.

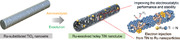

## 1 Introduction

Exsolution has received significant attention owing to its effectiveness in stabilizing metal nanostructures in solid matrices.^[^ ^1^ ^]^ In this approach, multimetallic oxides are heated in a reductive atmosphere, leading to the diffusion of highly reducible component metal ions onto the surface of a solid substrate and the simultaneous formation of zero‐valent metal nanoparticles.^[^ ^1^ ^−^ ^3^ ^]^ The remaining part of the metal nanoparticles remains embedded in the oxide substrate, enabling robust anchoring of the metal species.^[^ ^4^, ^5^ ^]^ As metal nanostructures exhibit outstanding electrocatalytic properties,^[^ ^6^ ^−^ ^8^ ^]^ the exsolution of metal nanoparticles is presumed to be effective for realizing efficient electrocatalysts.^[^ ^5^, ^9^, ^10^ ^]^ Notably, exsolved metal nanoparticles exhibit robust adhesion to inorganic substrates, which imparts excellent electrochemical and structural stability during electrocatalytic reactions, owing to the effective prevention of particle agglomeration.^[^ ^5^, ^11^, ^12^ ^]^ Recently, exsolved metal nanoparticles‐based hybrid materials have been utilized as electrocatalysts.^[^ ^13^, ^14^ ^]^ To further enhance the electrocatalytic activity of these hybrids, controlling the electrical conductivity of the exsolution substrate is supposed to be crucial.^[^ ^15^ ^]^ This is because metal oxide substrates have a low electrical conductivity, detrimentally affecting the electrocatalytic performance of the immobilized metal nanoparticles. Therefore, improving the electrical conductivity of the exsolution matrix is necessary to develop high‐performance electrocatalysts. As metal–nitrogen bonds have a higher covalency than metal−oxygen bonds,^[^ ^16^ ^]^ metal nitrides are expected to show higher electrical conductivity than metal oxides. In one instance, the electrical resistivity of TiN is reported to be ≈25 µΩ cm, which is much lower than that of TiO_2_ (≈10^5^−10^6^ µΩ cm).^[^ ^17^, ^18^ ^]^ Hence, the metal nitride is a more suitable substrate for electrocatalyst applications. However, compared to metal oxides, metal nitrides suffer from lower thermal stability and limited compositional tunability, which hinder their direct use as an exsolution matrix.^[^ ^19^ ^]^ Recently, the heat treatment of metal oxides under an NH_3_ flow at elevated temperatures was reported to induce a phase transition of semiconductive metal oxides to metallic metal nitrides.^[^ ^20^, ^21^ ^]^ Simultaneously, the reductive atmosphere created by the NH_3_ flow was expected to allow the exsolution of noble‐metal ions to metal nanoparticles at elevated temperatures. Therefore, the ammonolysis of noble metal‐containing multimetallic oxides could effectively stabilize noble‐metal nanoparticles in conductive metal nitride substrates. 1D nanowires of titanium ruthenium oxide (Ti_1−x_Ru_x_O_2_) are considered a promising material for nitridation‐driven exsolution. The ammonolysis of TiO_2_ nanowires results in the aliovalent substitution of three O^2−^ ions with two N^3−^ ions. This induces the formation of abundant anion vacancies, yielding the 1D holey TiN nanotubes with many crystal defects.^[^ ^20^, ^21^ ^]^ Therefore, the heat treatment of Ti_1−x_Ru_x_O_2_ nanowires under NH_3_ flow is expected to induce the exsolution of Ru metal nanoparticles embedded in holey TiN nanotubes.^[^ ^5^, ^20^ ^]^ The resulting immobilization of noble‐metal nanoparticles in porous conductive TiN nanotubes enhances both the mass and electron transport properties, optimizing the electrocatalytic activity. Despite the many merits of immobilizing metal nanoparticles in a metal nitride matrix, to the best of our knowledge, no studies have investigated the ammonolysis‐driven exsolution of zero‐valent metal nanoparticles embedded in conductive metal−nitride substrates.

In this study, we devised a nitridation‐driven exsolution approach, in which 1D multimetallic Ti_1−x_Ru_x_O_2_ nanowires are treated with NH_3_ at elevated temperatures. The obtained holey TiN nanotubes embedded with the exsolved Ru nanoparticles were employed as electrocatalysts for a hydrogen evolution reaction (HER) to verify the effectiveness of this synthetic strategy in producing high‐performance stable electrocatalysts. To elucidate the underlying mechanism of this synthetic strategy, the interfacial coupling between the Ru nanoparticles and holey 1D TiN nanotubes was systematically investigated using spectroscopic and theoretical methods, including in situ Raman spectroscopy and density functional theory (DFT) calculations.

## 2 Results and Discussion

### 2.1 Evolution of the Crystal Structure and Morphology of Ti_1−x_Ru_x_O_2_ Nanowires upon Ammonolysis

**Figure 1:**
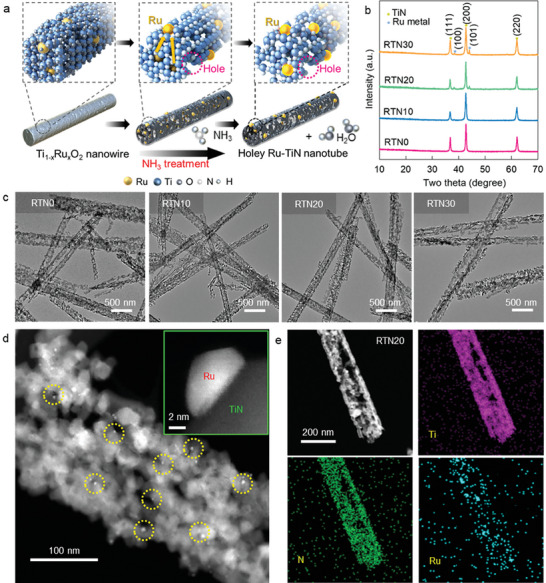
a) Scheme for a nitridation‐driven exsolution route to holey Ru─TiN nanotubes. b) Powder XRD, c) TEM images of holey Ru─TiN nanotubes. d) STEM and enlarged image (inset) and e) EDS−elemental mapping data of RTN20.

As shown in **Figure** **1**a, Ti_1−x_Ru_x_O_2_ nanoparticle precursors were prepared by hydrolyzing titanium isopropoxide in the presence of RuCl_3_·H_2_O at 85 °C for 8 h. The subsequent hydrothermal treatment, annealing in ambient atmosphere, and hydrothermal treatment of Ti_1−x_Ru_x_O_2_ nanoparticles at 200 °C for 12 h in a 10 m NaOH solution yielded 1D multimetallic Ti_1−x_Ru_x_O_2_ nanowires,^[^ ^20^, ^22^ ^]^ which were employed as precursors for nitridation−exsolution. To study the influence of cation composition, we synthesized 1D Ti_1−x_Ru_x_O_2_ nanowires with x = 0, 0.1, 0.2, and 0.3, which were named RTO0, RTO10, RTO20, and RTO30, respectively. The resulting 1D Ti_1−x_Ru_x_O_2_ nanowires were subjected to ammonolysis to induce one‐pot nitridation−exsolution. Exsolved Ru─TiN nanotubes were obtained through the heat treatment of Ti_1−x_Ru_x_O_2_ nanowires at 900 °C for 3 h under a flow of NH_3_ gas. The resulting Ru─TiN nanotubes were denoted as RTN0, RTN10, RTN20, and RTN30.

The structural modifications of Ti_1−x_Ru_x_O_2_ induced by the Ru substitution and ammonolysis were characterized via powder X‐ray diffraction (XRD) technique. All Ti_1−x_Ru_x_O_2_ precursors (0 ≤ x ≤ 0.3) exhibit XRD peaks characteristic of the layered trititanate phase, indicating the presence of a layered trititanate structure with a space group of C12/M1 (Figure S1, Supporting Information) in the materials.^[^ ^23^ ^−^ ^25^ ^]^ As evident from Table S1 (Supporting Information), the unit cell volume of the Ti_1−x_Ru_x_O_2_ materials increases with increasing Ru content. This volume increase can be attributed to the incorporation of larger Ru^4+^ ions (r(Ti^4+^) = 0.61 Å; r(Ru^4+^) = 0.76 Å) in the Ti_1−x_Ru_x_O_2_ lattice.^[^ ^26^ ^]^ The Ti_1−x_Ru_x_O_2_ nanowires have a 1D morphology, as verified using field emission‐scanning electron microscopy (FE‐SEM). As can be observed from Figure S2 (Supporting Information), all Ti_1−x_Ru_x_O_2_ materials have highly anisotropic 1D nanowire shapes.

After ammonolysis, all materials exhibit well‐defined peaks related to the TiN structure (Figure 1b), demonstrating the structural transformation from layered trititanate to titanium nitride phase. While the Ru‐unsubstituted RTN0 shows no Ru‐metal‐related XRD peaks, both RTN20 and RTN30 display weak peaks characteristic of the (100) and (101) planes of Ru metal, highlighting the exsolution of Ru^4+^ ions into elemental Ru nanoparticles. The low Ru content in RTN10 prevents the detection of Ru metal‐related reflections from the material. In contrast to the TiN phase, no ruthenium nitride is present in the nitridated Ru─TiN materials. To verify no formation of the ruthenium nitride phase during the ammonolysis, we performed the nitridation experiment for RuO_2_ material. As presented in Figure S3 (Supporting Information), the ammonolysis of RuO_2_ at the same condition is found to produce Ru metal rather than ruthenium nitride. The reductive formation of neutral Ru metal phase during the ammonolysis could be attributed to the high standard reduction potential of Ru metal. The FE‐SEM analysis confirms that ammonolysis significantly alters the crystal morphology of Ti_1−x_Ru_x_O_2_. As shown in Figure S4 (Supporting Information), the ammonolysis of 1D Ti_1−x_Ru_x_O_2_ nanowires changes the morphology of the nanowires to hollow nanotubes containing surface holes, indicating the formation of holey TiN nanotubes. Figure 1c,d shows the transmission electron microscopy (TEM) and scanning transmission electron microscopy (STEM) images of the as‐obtained Ru─TiN nanotubes. Small Ru nanoparticles are immobilized on the surface of the TiN nanotubes, as indicated by the small bright spots in the holey TiN nanotube matrix. As shown in Figure S5 (Supporting Information), the thickness of TiN nanotubes in all RTN materials ranges from ≈60 to 330 nm and the particle size of exsolved Ru nanoparticles in all RTN ranges from ≈2 to 25 nm. Increasing the Ru content in RTN materials leads to a higher degree of exsolution. Energy‐dispersive spectroscopy (EDS)−elemental mapping analysis revealed a homogeneous distribution of Ru, Ti, and N (Figure 1e) within the nanotubes, offering conclusive evidence for the formation of TiN nanotubes anchored with Ru nanoparticles.

The advantage of ammonolysis‐driven exsolution in enhancing the porosity of 1D Ru─TiN nanotubes was demonstrated by N_2_ adsorption−desorption isotherm measurements. As shown in Figure S6 (Supporting Information), all Ru─TiN nanotubes exhibit a similarly featured Brunauer−Deming−Deming−Teller (BDDT) type IV isotherm and an H3‐type hysteresis loop, reflecting their mesoporous nature.^[^ ^27^, ^28^ ^]^ The Brunauer−Emmett−Teller (BET) surface areas of RTN0, RTN10, RTN20, and RTN30 are 19, 27, 32, and 40 m^2^ g^−1^, respectively. This indicates that porosity increases with an increase in the amount of exsolved Ru.

### 2.2 Bonding Characters of the Ti_1−x_Ru_x_O_2_ Nanowires and the Holey Ru─TiN Nanotubes

**Figure 2:**
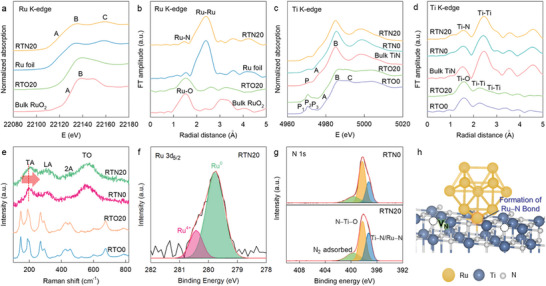
a) Ru K‐edge XANES, b) Ru K‐edge FT‐EXAFS, c) Ti K‐edge XANES, d) Ti K‐edge FT‐EXAFS, e) micro‐Raman, f) Ru 3d_5/2_ XPS, and g) N 1s XPS data of RTO nanowires and exsolved RTN nanotubes. h) Schematic illustration of the formation of interfacial Ru^4+^─N─Ti^3+^ bonds in exsolved RTN nanotubes.

We investigated the evolutions of the electronic and local crystal structures of 1D Ti_1−x_Ru_x_O_2_ nanowires following the NH_3_ treatment using X‐ray absorption near‐edge structure (XANES) and extended X‐ray absorption fine structure (EXAFS) analyses at the Ru and Ti K‐edges. **Figure** **2**a shows the Ru K‐edge XANES spectrum. All the investigated materials display several main‐edge peaks (A, B, and C) attributed to the dipole‐allowed 1s → 5p transitions. The edge energy of RTO20 is close to that of RuO_2_, indicating the stabilization of the tetravalent Ru^4+^ ions.^[^ ^5^, ^29^ ^]^ After the ammonolysis of RTO20, its overall spectral features change to zero‐valent Ru^0^ metal‐like features, providing convincing proof for the exsolution of Ru^4+^ cations into metallic Ru nanoparticles. Ru K‐edge EXAFS analysis revealed that the ammonolysis was accompanied by local structural transition. As can be observed from Figure 2b, RTO20 exhibits typical Fourier transform (FT) features exhibited by a layered trititanate phase,^[^ ^30^ ^]^ clarifying the substitution of Ru ions into the Ti sites of the trititanate lattice. Conversely, RTN20 exhibits an intense FT peak at ≈2.4 Å, which is ascribed to the Ru─Ru bonding pair, as observed in the Ru metal reference. This finding substantiates the formation of Ru nanoparticles via the ammonolysis‐driven exsolution of Ru^4+^ ions. RTN20 exhibits weaker and broader Ru─Ru‐related peaks than Ru metal, reflecting the formation of smaller Ru nanoparticles after exsolution. In addition, RTN20 exhibits a small but distinct FT peak at ≈1.6 Å, strongly indicating the presence of Ru─N bonds between Ru nanoparticles and TiN nanotubes. The nonlinear curve‐fitting analysis (**Table** **1**; Figure S7a, Supporting Information) reveals that the coordination number of the Ru─Ru bond pair in RTN20 is 8.9, which is notably smaller than that of the Ru foil reference (coordination number = 12), clearly indicating the creation of nanosized Ru particles in this material. The additional Ru─N coordination shell exhibits a coordination number of ≈1.3, attesting to the distinct chemical interaction between the exsolved Ru nanoparticles and TiN matrix. Figure 2c shows the results of the Ti K‐edge XANES analysis. Both RTO0 and RTO20 exhibit spectral features characteristic of the layered trititanate phase, such as pre‐edge peaks (P_1_, P_2_, and P_3_) ascribed to quadrupole‐allowed 1s → 3d transitions and main‐edge peaks (A, B, and C) ascribed to the dipole‐allowed 1s → 4p transitions.^[^ ^31^, ^32^ ^]^ Both materials display very similar edge energies, strongly indicating the stabilization of the tetravalent Ti^4+^ oxidation state.

**Table 1:** Structural parameters of holey Ru─TiN nanotubes, TiN nanotubes, bulk TiN, and Ru metal determined by Ti K‐edge and Ru K‐edge EXAFS analyses.

Material	Bonding pair	Coordination number	R [Å]	ΔE [eV]	𝜎^2^ [10^−3^ x Å^2^]
RTN20	Ti─N	4.7	2.08	3.72	2.24
	Ru─Ru	8.9	2.67	2.16	2.54
	Ru─N	1.3	2.12	1.55	8.71
RTN0	Ti─N	5.5	2.08	4.18	4.39
Bulk TiN	Ti─N	6.0	2.09	3.80	3.80
Ru metal	Ru─Ru	12.0	2.68	2.56	3.34

The ammonolysis of both RTO0 and RTO20 induces significant changes in their spectra to resemble the spectrum of TiN with a marked red‐shift of the edge energy, supporting a structural transition into a trivalent Ti^3+^N phase. Such structural modifications following Ru substitution and ammonolysis were confirmed by Ti K‐edge EXAFS analysis. As can be observed from Figure 2d, both RTO0 and RTO20 exhibit typical FT features of the layered trititanate phase, including peaks ascribed to Ti─O, edge‐shared Ti─Ti, and corner‐shared Ti─Ti bonds, respectively. This indicates that the layered trititanate structure is maintained after Ru substitution. The ammonolysis of both RTN0 and RTN20 induces remarkable spectral changes to TiN‐type FT features, such as peaks ascribed to Ti─N and Ti─Ti bonds at ≈1.6 and 2.5 Å, respectively, confirming the formation of TiN structure. As evident from Table 1 and Figure S7b (Supporting Information), the curve fitting analysis revealed that the Ti─N bond of RTN20 has a smaller coordination number (4.7) than Ru‐free RTN0 (5.5) and bulk TiN (6.0), indicating a higher concentration of nitrogen vacancies in RTN20. This finding provides convincing evidence for the creation of crystal vacancies during the exsolution of Ru ions onto the surface region.

The creation of nitrogen vacancies following the ammonolysis‐driven exsolution is further corroborated by the results of micro‐Raman spectroscopy. As evident from Figure 2e, all the Ti_1−x_Ru_x_O_2_ precursors exhibit typical phonon lines of the layered titanate phase,^[^ ^33^ ^]^ whereas the RTN materials show phonon lines characteristic of the TiN phase, indicating a nitridation‐induced phase transition to rocksalt‐type titanium nitride structure.^[^ ^20^, ^21^, ^34^ ^]^ The increase in Ru content led to gradual displacement of the acoustic phonon line at ≈200 cm^−1^ toward higher wavenumbers. Because the energy of this Raman peak is proportional to the concentration of nitrogen vacancies,^[^ ^35^ ^]^ the increase in the phonon energy with increasing Ru content can be interpreted as further evidence for the creation of nitrogen vacancies caused by the exsolution of Ru nanoparticles.

The formation of interfacial bonding of the exsolved Ru nanoparticles with holey TiN nanotubes was cross‐confirmed via X‐ray photoelectron spectroscopy (XPS). Due to the overlap between the Ru 3d_3/2_ and C 1s signals, only the Ru 3d_5/2_ peak could be analyzed by curve fitting analysis to characterize the surface bonding nature of RTN20. Although the EXAFS result indicates that the oxidation state of ruthenium in RTN20 is Ru^0^, the Ru 3d_5/2_ XPS analysis of RTN20 clearly demonstrates the co‐existence of two types of Ru^0^ and Ru^4+^ species, indicating the formation of interfacial Ru^4+^─N─Ti^3+^ bond (Figure 2f). Since the XPS technique is sensitive to surface property, the observed Ru─N component could be interpreted as a result of interface bonding between Ru metal and TiN substrate. In the N 1s region (Figure 2g), the Ru‐exsolved RTN20 displays a larger spectral weight for the low energy component corresponding to metal−nitrogen bonds (i.e., Ti─N/Ru─N) with respect to the Ru‐free RTN0 material. The observed spectral alteration can be interpreted as further proof supporting the additional formation of interfacial Ru^4+^─N^3−^ bonds. All spectroscopic results clearly demonstrate that the ammonolysis of 1D Ti_1−x_Ru_x_O_2_ nanowires induces the exsolution of metallic Ru nanoparticles with a phase transformation from layered trititanate to titanium nitride and the formation of Ru^4+^─N─Ti^3+^ bonds and nitrogen vacancies in the TiN nanotube matrix (Figure 2h).

### 2.3 Electrocatalyst Functionalities of the Holey Ru─TiN Nanotubes

**Figure 3:**
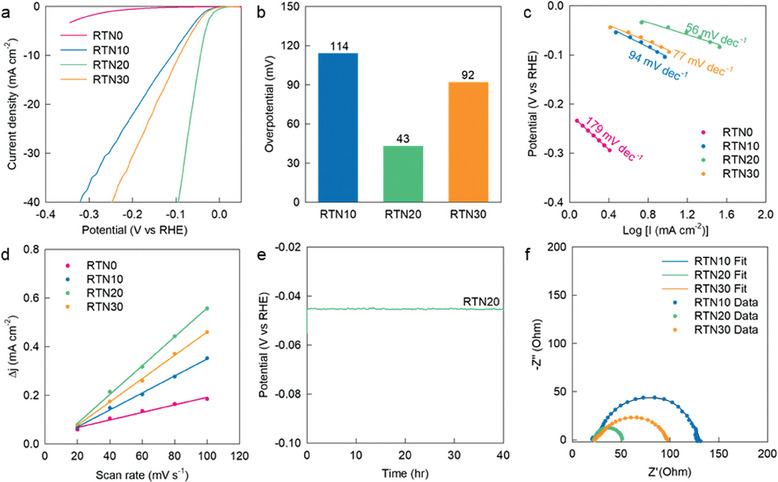
a) LSV curves, b) overpotentials, c) Tafel plots, d) ECSA, e) stability data, and f) Nyquist plots for exsolved RTN nanotubes.

The effectiveness of the nitridation−exsolution process in increasing electrocatalytic activity was assessed by employing 1D Ru─TiN nanotubes and precursor Ti_1−x_Ru_x_O_2_ nanowires as HER electrocatalysts. As illustrated by the linear sweep voltammetry (LSV) curves (**Figures** **3**a; Figure S8, Supporting Information), compared to Ti_1−x_Ru_x_O_2_ precursors, all Ru─TiN nanotubes deliver superior HER electrocatalyst performance with lower overpotentials and larger current densities. This underscores the high efficacy of the ammonolysis strategy in realizing high‐performance HER electrocatalysts.

The Ru‐exsolved RTN10, RTN20, and RTN30 nanotubes exhibit higher electrocatalytic activities than the Ru‐free RTN0, confirming the contribution of the exsolved Ru species in improving the HER activity. Among the Ru‐exsolved TiN nanotubes under investigation, RTN20 exhibits the optimum HER performance, with the lowest overpotential of 43 mV at 10 mA cm^−2^, which is smaller with respect to those of the other homologs (Figure 3b). This result highlights that a Ru content corresponding to x = 0.2 is the optimal concentration. The significant influence of Ru concentration on the electrocatalytic HER kinetics was verified by the calculation of Tafel slopes. As shown in Figure 3c and **Table** **2**, RTN20 exhibits a lower Tafel slope of 56 mV dec^−1^ than the other homologs, confirming that the incorporation of Ru ions at x = 0.2 optimizes the HER kinetics of Ru─TiN materials. In addition, the effects of reaction temperature and time on the crystal morphology and HER activity were examined. As presented in Figures S9 and S10 (Supporting Information), the lowering of the reaction temperature below 900 °C hinders the formation of surface holes, whereas the shortening or elongation of reaction time from 3 h leads to the deterioration of HER activity. These results clearly demonstrate that the ammonolysis at 900 °C for 3 h is an optimal condition to optimize the electrocatalyst performance.

**Table 2:** Summary of hydrogen evolution reaction activity of holey Ru─TiN nanotubes, TiN nanotube, and Ru‐deposited RTN20‐D in acidic electrolyte.

Material	η^10^ [mV]	Tafel slope [mV dec^−1^]	ECSA [mF cm^−2^]	R_ct_ [Ω]
RTN0	–	179	1.95	388
RTN10	114	94	4.36	114
RTN20	43	56	7.01	41
RTN30	92	77	5.93	88
RTN20‐D	87	81	8.49	98

By measuring the electrochemical double‐layer capacitance, the electrochemically active surface areas (ECSAs) of the Ru─TiN nanotubes were estimated to probe the evolution of the surface activity. As shown in Figure 3d and Table 2, the RTN20 exhibits an ECSA of 7.01 mF cm^−2^, which is higher than those of RTN0, RTN10, and RTN30, underscoring the benefit of optimal Ru concentration. As evident from Figure 3e, RTN20 exhibits excellent electrocatalyst durability with negligible capacity degradation (≈0.4% for 40 h), highlighting the improvement in durability achieved by the exsolution of an optimal amount of Ru substituents. The improved stability of Ru‐exsolved RTN20 was further confirmed by monitoring its morphological evolution during the HER activity test. As depicted in Figure S11 (Supporting Information), the Ru‐exsolved RTN20 retains a 1D nanotube morphology without notable particle size change and particle aggregation after the HER activity test, confirming its high structural stability. Electrochemical impedance spectroscopy (EIS) analysis provided additional evidence for the advantage of the nitridation−exsolution process in optimizing interfacial charge transfer properties. As shown in Figure 3f, at an applied potential of −0.1 V (vs. RHE), the Ru‐exsolved RTN10, RTN20, and RTN30 display a semicircle in the mid‐high frequency range. The diameter of the semicircle is inversely proportional to the charge transfer resistance (R_ct_). Conversely, the Ru‐free RTN0 exhibits a large semicircle, indicating its poor charge transfer kinetics (Figure S12, Supporting Information). As evident from the non‐linear fitting analysis of the EIS data (Table 2), RTN20 displays a smaller R_ct_ value of 41 Ω than the other homologs, highlighting the optimization of interfacial charge transport during the nitridation−exsolution process at the optimal Ru content. As can be observed from Table S2 (Supporting Information), the overall HER performance of RTN20 (the η^10^ value) is superior or comparable to those of recently published state‐of‐the‐art Ru metal nanoparticle catalysts.

### 2.4 Relative Efficiency of Exsolution and Deposition Methods in Regulating the HER Activity of the Holey Ru─TiN Nanotubes

**Figure 4:**
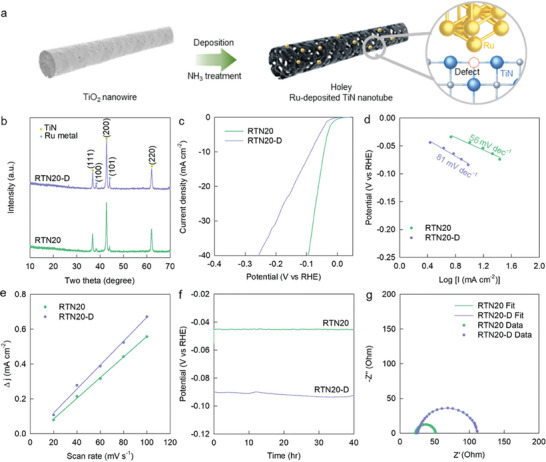
a) Scheme for the formation of holey Ru‐deposited TiN nanotubes. b) Powder XRD, c) LSV, d) Tafel plots, e) ECSA, f) stability data, and g) Nyquist plots for Ru‐deposited RTN20‐D.

To probe the superior advantage of the exsolution strategy in enhancing the electrocatalytic activity over the conventional surface deposition strategy, Ru‐deposited holey TiN nanotubes were synthesized with an identical composition to RTN20 through the nitridation of TiO_2_ nanowires and subsequent deposition of Ru nanoparticles, as shown in **Figure** **4**a. The resulting material was designated as RTN20‐D. The stabilization of Ru metal nanoparticles in the RTN20‐D material is confirmed by powder XRD analysis, which shows Bragg reflections corresponding to the cubic TiN and metallic Ru phases (Figure 4b). Compared to the exsolved RTN20, RTN20‐D displays higher intensity Ru metal‐related peaks, reflecting the higher crystallinity of the deposited Ru metal species. The STEM image of RTN20‐D clearly shows Ru metal nanoparticles deposited on the surface of the holey TiN nanotubes with a Ru particle size of ≈2−23 nm (Figure S13, Supporting Information).

The relative efficiency of the exsolution method over the deposition method in realizing efficient electrocatalysts was studied by comparing the HER performance of RTN20‐D with that of RTN20. As illustrated by the LSV data (Figure 4c,d), compared to the Ru‐exsolved RTN20, the Ru‐deposited RTN20‐D delivers inferior HER functionality (i.e., overpotential = 87 mV, Tafel slope = 81 mV dec^−1^). This result provides convincing evidence for supporting the higher efficacy of the nitridation‐driven exsolution strategy in improving the electrocatalytic activity compared to that of the conventional deposition method. Despite a lower electrocatalytic activity, RTN20‐D shows a slightly larger ECSA of 8.49 mF cm^−1^ than that of the exsolved RTN20 sample (7.01 mF cm^−1^), which can be attributed to the surface roughening due to the surface deposition of Ru nanoparticles (Figure 4e). RTN20‐D exhibits a decay of ≈1.4% for 40 h, which is higher than that of RTN20 (≈0.4%) (Figure 4f), indicating the poorer stability of the deposited material. Moreover, the STEM image of RTN20‐D shows the significant agglomeration of Ru nanoparticles after the stability test, which is in stark contrast to the RTN20 material maintaining its structure without notable particle agglomeration (Figures S11 and S14, Supporting Information). This result confirms that the RTN20 prepared with the exsolution method is more stable than the RTN20‐D prepared with the deposition method.

To examine the effect of the nitridation–exsolution methodology on the charge transport behavior, the EIS curve of RTN20‐D was compared with that of RTN20. As shown in Figure 4g, the radius of the semicircle is much larger for RTN20‐D than for RTN20, confirming the inferior charge‐transfer kinetics of the deposited material. Using the least squares fitting analysis allowed us to determine the R_ct_ of RTN20‐D as 98 Ω, which was much higher than that of exsolved RTN20 (41 Ω). This confirms that the nitridation−exsolution strategy is more efficient in improving the interfacial charge transfer behavior than the conventional deposition process.

**Figure 5:**
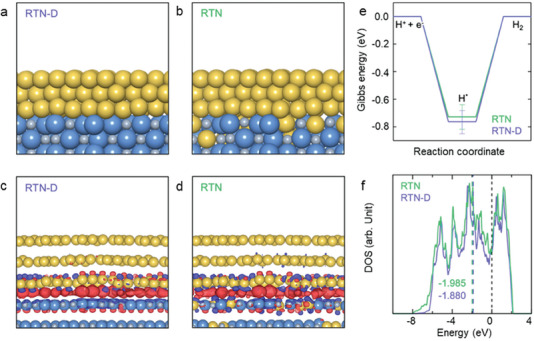
DFT‐optimized slab model of a) Ru‐deposited Ru─TiN nanotube (RTN‐D) and b) Ru‐exsolved Ru─TiN nanotube (RTN). Charge density difference plot of c) RTN‐D and d) RTN. The isosurface levels are taken as ± 0.01 *e* Bohr^−3^. Red and blue regions indicate charge accumulation and depletion, respectively. e) DFT‐calculated average HER energy profiles. The bar brackets the minimum and maximum values calculated in DFT (Figure S15 and Table S3, Supporting Information). f) Density of state (DOS) of RTN‐D and RTN (with the *d*‐band levels indicated). The Ru, Ti, and N atoms are depicted as yellow, blue, and gray spheres, respectively.

To further understand the differences between the exsolved Ru nanostructures in RTN and the deposited Ru nanoparticles in RTN‐D, DFT calculations were performed using periodic slab models to investigate the influence of their interfacial energetics and electronic structures on their HER performances. Based on previous studies that reported that Ru(0001) is the most thermodynamically stable surface facet of the hexagonal close‐packed (hcp) Ru phase,^[^ ^36^, ^37^ ^]^ we constructed an atomic interface model of RTN‐D (**Figure** **5**a) by considering a few layers of Ru(0001) supported on pristine TiN(001). Both RTN and RTN‐D exhibit fairly similar XRD profiles (Figure 4b). Hence, the interfaces of the RTN nanostructures were modeled using those of RTN‐D, in which Ru atoms are randomly substituted at the Ti sites in the TiN(001) support, as illustrated in Figure 5b. As shown in Figure 5c,d, the charge density difference plots demonstrate that, for both the RTN and RTN‐D interface models, charge accumulation (in red) is highly concentrated at the interface regions, suggesting that the TiN matrix and Ru atoms are chemically bonded.

To further quantify this chemical interaction between the TiN matrix and Ru atoms, we defined and calculated the interfacial energy, which is a critical parameter in evaluating the thermodynamic stability of a supported nanocatalyst.^[^ ^38^ ^−^ ^41^ ^]^ The area‐normalized interfacial energies of RTN‐D and RTN are −0.20 and −0.22 eV Å^−2^, respectively, indicating that RTN has potentially a higher interfacial stability than RTN‐D. This result aligns with our experimental results, wherein the RTN catalyst exhibits higher stability (Figure 4f). In addition, the HER Gibbs energy $\Delta G_{H^{*}}$ of these two Ru/TiN interface models was calculated to better understand their relative HER catalytic activity at the atomic scale (Figure 5e). For the adsorption of H on the Ru(0001) surface, two hollow hcp and two fcc‐hollow sites were considered for both RTN and RTN‐D (Figure S15, Supporting Information). During geometry optimization, the hydrogen atom was found to be unstable at the atop site and preferentially moved to a neighboring hollow site. Hence, no atop site adsorption information is available. For both RTN and RTN‐D, the adsorption of H at the fcc hollow sites is thermodynamically more favorable, which is consistent with the reported behavior of H adsorption on pristine hcp Ru(0001).^[^ ^41^ ^]^ The average $\Delta G_{H^{*}}$ of H on RTN and RTN‐D is calculated to be −0.728 and −0.763 eV, respectively. The negative sign implies that H adsorption is thermodynamically favorable in both cases. Notably, as a chemical descriptor, $\Delta G_{H^{*}}$ should be close to zero for a better HER performance wherein the H_2_ molecule is desorbed more favorably. Therefore, based on our DFT Gibbs energy calculations, we conclude that the RTN model outperforms RTN‐D during a HER. More details of the calculations of the site‐dependent $\Delta G_{H^{*}}$ values are tabulated in Table S3 (Supporting Information).

To elucidate the electronic structure underlying the comparative catalytic activity of RTN and RTN‐D, the projected density of states (PDOS) was calculated for both interface models, as shown in Figure 5f. It is well‐known that the electronic structure of a supported nanocatalyst can be modulated by varying and engineering the substrate support.^[^ ^42^, ^43^ ^]^ The PDOS plots reveal that the *d*‐bands of Ru in RTN are left‐shifted compared to that in RTN‐D. Their relative reactivity for HER could be evaluated using the *d*‐band center, which is a widely used chemical descriptor.^[^ ^31^ ^]^ Hcp Ru surfaces are reported to exhibit a strong affinity for H (i.e., they have excessively high H adsorption energies).^[^ ^41^, ^44^, ^45^ ^]^ Therefore, as per the Sabatier principle, the strong chemical bonds between H and the Ru surface must be weakened to prevent overbinding of H during the HER. The left shift of the *d*‐band center of Ru indicates a chemical bond weakening, as more antibonding states are usually found near the Fermi level.^[^ ^46^ ^]^ The *d*‐band center of the Ru atoms in the RTN interface model (−1.985 eV) is calculated to be left‐shifted compared to that of the RTN‐D model (−1.880 eV), which is responsible for the higher efficiency of the RTN material during the HER process. In summary, the present DFT calculations provide a simple electronic structure model to elucidate the underlying mechanism behind the higher interfacial stability and superior HER performance of the exsolved Ru atoms in RTN.

### 2.5 Evolution of the Structural, Morphological, and Chemical Features of Holey Ru─TiN Nanotubes During the HER Process

**Figure 6:**
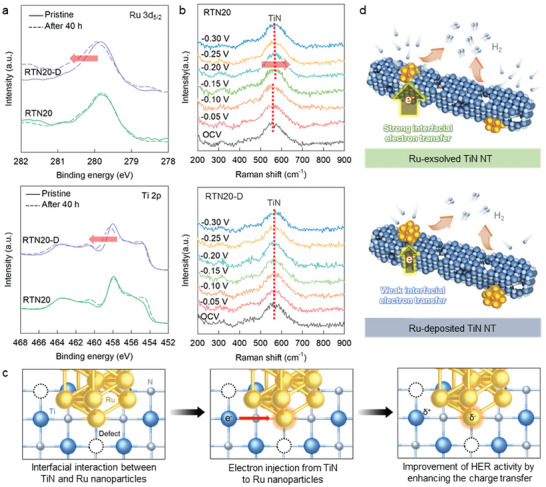
a) Ru 3d_5/2_ and Ti 2p XPS data of RTN and RTN20‐D after the HER test. b) In situ Raman analyses for RTN20 and RTN20‐D during the HER test. c) Scheme for interfacial electron injection from TiN nanotubes to Ru nanoparticles. d) Scheme for the interfacial structure of holey Ru─TiN nanotubes and its effect on the HER process.

The benefit of exsolution in stabilizing the metal nanoparticles was verified by examining the XPS spectra of Ru‐exsolved RTN20 and Ru‐deposited RTN20‐D before and after the HER activity test. As shown in **Figure** **6**a, the exsolved RTN20 exhibits only negligible spectral changes in the Ru 3d_5/2_ XPS data, which is in stark contrast to the marked variation observed for the deposited RTN20‐D. This confirms the benefits of the exsolution process in stabilizing Ru nanoparticles. In addition, significantly lesser alterations in Ti 2p data are observed for RTN20 than for RTN20‐D, attesting to the improvement in RTN materials caused by the exsolution process. The promoted interfacial charge transfer between the exsolved materials and the reactant during the HER minimizes charge accumulation in the catalyst materials, which is responsible for the increased stability of the exsolved RTN20‐D.

To experimentally elucidate the underlying mechanism behind the enhanced HER activity of the exsolved Ru nanoparticles, in situ Raman spectra of the Ru‐exsolved RTN20 and Ru‐deposited RTN20‐D were obtained during the HER. As shown in Figure 6b, both materials exhibit the characteristic phonon lines of the TiN lattice at ≈320, 450, and 580 cm^−1^. RTN20‐D exhibits no significant shift of Raman peaks during the HER process. By contrast, the TiN‐related phonon line exhibited by RTN20 at 580 cm^−1^ exhibits a notable shift toward the high energy side upon the application of the reduction potentials (>−0.15 V). This notable peak shift indicates that the Ti^─^N bond distance decreases with increasing electrical potential. This is attributable to the promotion of electron transfer from the TiN nanotubes into the exsolved Ru nanoparticles, as illustrated in Figure 6c. The absence of a notable peak shift for RTN20‐D confirms the weak electronic coupling of the deposited Ru nanoparticles with the TiN substrate.^[^ ^5^ ^]^ The enhanced electrocatalytic performance of the immobilized Ru species following exsolution can be primarily attributed to the strong interfacial electronic coupling between the Ru nanoparticles and conductive TiN nanotubes.

The impact of the exsolution method on the chemical stability of the metal nanoparticles was verified by analyzing the concentration of dissolved metal ions in the catalysts after the stability test. As presented in Table S4 (Supporting Information), the exsolved RTN20 exhibits no Ru dissolution, whereas the deposited RTN20‐D homolog experiences significant Ru dissolution, indicating that the exsolved Ru nanoparticles are much more stable than the deposited Ru nanoparticles during the HER. Finally, as depicted in Figure 6d, the obtained Ru─TiN nanotubes are composed of holey TiN nanotubes embedded with Ru nanoparticles with an increased number of nitrogen vacancies. Robust Ru^4+^─N─Ti^3+^ bonds are formed in the interfacial regions composed of the Ru metal nanoparticles embedded in holey TiN nanotubes. Additionally, such significant interfacial bonding plays a pivotal role in expediting electron transfer between the TiN nanotubes and the nanosized Ru particles, which contributes to the improvement in the HER performance following nitridation‐driven exsolution.^[^ ^47^, ^48^ ^]^ At the same time, the robust anchoring of the Ru nanoparticles on the TiN nanotube enhances the durability of the Ru particles during the HER process. Therefore, the generation of significant interfacial interactions, as well as the increase in porosity and crystal defects, is found to be crucial in improving the electrocatalyst functionality of the Ru─TiN nanotubes. As evidenced by the EIS and in situ Raman results, the nitridation‐driven exsolution process promotes the interfacial electron transfer to the immobilized Ru nanoparticles, resulting in the further enhancement of the HER performance.

## 3 Conclusion

In the present study, we developed a versatile nitridation‐driven exsolution approach to realize stable high‐performance electrocatalysts, in which noble metal nanoparticles are embedded in a conductive metal nitride matrix. The ammonolysis of multimetallic 1D Ti_1−x_Ru_x_O_2_ nanowires at elevated temperatures led to the exsolution of Ru^4+^ substituent ions onto metallic Ru nanoparticles as well as the simultaneous phase transformation of TiO_2_ nanowires into holey TiN nanotubes, yielding 1D Ru─TiN nanotubes. The intimate anchoring of exsolved Ru nanoparticles onto the nanotubes resulted in the introduction of nitrogen vacancies into the TiN nanotubes and the creation of interfacial Ru^4+^─N─Ti^3+^ bonds. The obtained Ru‐exsolved holey TiN nanotubes delivered superior electrocatalytic HER functionality over the Ru‐deposited holey TiN nanotubes and precursor Ti_1−x_Ru_x_O_2_ nanowires. The benefit of this single‐step nitridation−exsolution process can be ascribed to the improved kinetics of interfacial charge transfer and the increased electron density in the Ru nanoparticles caused by enhanced charge transfer with the conductive TiN nanotubes, as evidenced by in situ Raman spectroscopy and EIS results. DFT calculations clearly demonstrated that the effective interfacial interaction between the exsolved Ru nanoparticles and the TiN nanotubes lowered the *d*‐band center energy, which optimized the adsorption−desorption behavior of hydrogen. The Ru nanoparticles in the Ru‐exsolved TiN nanotube exhibited a smaller hydrogen binding energy, attesting to their superior HER performance compared to that of the Ru‐deposited homolog. Nitridation‐driven exsolution was effective in improving the electrochemical stability of the exsolved Ru metal nanoparticles, owing to strong interfacial chemical interactions with the embedded TiN matrix. In addition to electrocatalytic applications, the obtained metal nanoparticle‐anchored metal nitride nanocomposites are expected to show excellent performance as electrodes in metal−sulfur and metal−air batteries, sensors, etc. Considering the high electrical conductivity of many metal nitrides such as NbN, TaN, and MoN, the ammonolysis‐driven exsolution strategy developed in this study could provide valuable opportunities to synthesize diverse functional metal nanoparticles embedded in conductive metal nitride matrices. Furthermore, this study suggests the possibility of developing new exsolution‐based synthetic approaches to immobilize metal nanostructures in various conductive matrices such as metal phosphides and chalcogenides. This is because the heat treatment of multimetallic oxides under a controlled atmosphere like PH_3_ and H_2_S is expected to induce the phase transition of metal oxide to metal phosphides and sulfides and the simultaneous exsolution of metal nanoparticles. In the future, we plan to apply the present synthetic strategy to zero‐ and 2D nanostructured multicomponent metal oxides to realize multifunctional metal‐based composite materials for renewable energy technologies.

## 4 Experimental Section

### Material Preparation

1D Ti_1−x_Ru_x_O_2_ nanoparticles precursors were prepared by hydrolyzing titanium isopropoxide (99.999%) with RuCl_3_·H_2_O (≥95%) at 85 °C for 8 h, accompanied by hydrothermal treatment at 180 °C for 12 h.^[^ ^22^ ^]^ Further, the precipitate from the cooled autoclave was centrifuged and washed using deionized water and ethanol several times and dried overnight in an oven at 50 °C. Finally, the dried precipitate was annealed at 500 °C in air for 3 h to obtain Ti_1−x_Ru_x_O_2_ nanoparticles. The resulting nanoparticles were transferred to a 50 mL Teflon‐lined stainless autoclave and heated at 200 °C for 12 h in a 10 m NaOH solution. To examine the influence of Ru content on the HER activity of Ru─TiN nanotubes, Ti_1−x_Ru_x_O_2_ nanowires with x = 0, 0.1, 0.2, and 0.3 were synthesized. To synthesize holey Ru─TiN nanotubes, the ammonolysis of Ti_1−x_Ru_x_O_2_ nanowires was conducted at 900 °C for 3 h in an NH_3_ atmosphere at a flow rate of 100 mL min^−1^, resulting in the exsolution of Ru ions into Ru metal nanoparticles and the nitridation of TiO_2_ nanowires. Ru‐deposited TiN nanotubes were achieved by reaction of the Ru‐free TiO_2_ nanowires with RuCl_3_·H_2_O for 12 h. Briefly, the Ru‐free TiO_2_ nanowires were dispersed in distilled water and then mixed with RuCl_3_·H_2_O. The product was purified by washing with distilled water and then dried overnight in an oven at 50 °C. Ammonolysis was performed under the same conditions as Ru─TiN nanotubes.

### Material Characterization

The structural characterizations of Ti_1−x_Ru_x_O_2_ nanowires and Ru─TiN nanotubes were accomplished via powder XRD (Rigaku MiniFlex/Ultima IV, λ = 1.5418 Å, 25 °C). After the ammonolysis, the crystal morphologies and composite structures of the nanowires were examined using FE‐SEM (JEOL JSM‐7001F), TEM (JEOL‐F200), and STEM (JEOL‐ARM200F). The chemical compositions and elemental distributions of these materials were examined via EDS−elemental mapping with a TEM machine. The porosities and surface areas of the obtained materials were analyzed via N_2_ adsorption–desorption isotherm measurements at 77 K using a BELSORP‐miniX analyzer. Both the XANES and EXAFS spectra at the Ti and Ru K‐edges were collected at the 8C and 10C beamlines in the Pohang Accelerator Laboratory (PAL, Pohang, Korea). The energy of the obtained data was calibrated with reference to the spectra of Ti and Ru metal foils. The oxidation states of the component elements in the materials were monitored via XPS (Thermo VG, UK, Al Kα). All the XPS data were energy‐referenced with respect to the adventitious C 1s peak (binding energy = 284.8 eV). The evolution of the chemical bonding nature and oxidation state during the electrocatalytic HER was investigated using in situ Raman analysis. Raman spectra were collected using a Horiba Jobin Yvon LabRam Aramis instrument, which uses an Ar ion laser (λ = 514.5 nm) as the excitation source. To improve the signal‐to‐noise ratio in the Raman data, an Au‐nanoparticle‐based surface‐enhanced Raman scattering substrate was used to immobilize the electrocatalyst materials.

### Electrochemical Measurement

The electrocatalytic HER activity of the obtained materials was tested using a standard three‐electrode electrochemical cell. The catalyst ink was obtained by dispersing the catalyst material (3.5 mg), Vulcan XC‐72 (1.5 mg) and a 5 wt% Nafion solution (20 µL) in a mixed solvent of isopropanol/Milli‐Q water (1:4, v/v, 2.5 mL), which was sonicated for 5 h. The catalyst ink (10 µL) was then loaded onto a glassy carbon electrode (3 mm diameter) and dried in an oven at 50 °C. The electrolyte was prepared by thoroughly purging N_2_ gas into an aqueous solution of 0.5 m H_2_SO_4_. A graphite rod and saturated calomel electrode (SCE) were used as the counter and reference electrodes, respectively. LSV curves were obtained using an RRDE‐3A (ALS Co.) rotator and IVIUM analyzer at a scan rate of 5 mV s^−1^ and a rotating speed of 1600 rpm. The electrical potentials were transformed into reversible hydrogen electrode (RHE) values using the equation E_RHE_ = E_SCE_ + 0.256 V. The evolution of charge transport behavior following the exsolution was examined by measuring EIS data (IVIUM analyzer) at −0.1 V (V vs. RHE) in the frequency range of 0.1−100000 Hz. The concentrations of the dissolved metal ions during the HER‐activity tests were measured using inductively coupled plasma‐mass spectrometry (Agilent).

### DFT Calculations

The Vienna Ab initio Simulation Package (VASP) was utilized for performing the DFT calculations via the projector augmented (PAW) method.^[^ ^49^, ^50^ ^]^ The optB86b exchange‐correlation (*xc*) functional was chosen to accurately incorporate the long‐range van der Waals contributions self‐consistently.^[^ ^51^ ^]^ The kinetic energy cutoff for plane waves was set to 500 eV and the Γ‐centered **k**‐point grid spacing was set to 0.15 Å^−1^. We constructed periodic slab models using the CellMatch code,^[^ ^52^ ^]^ combining three atomic layers of rocksalt TiN(001) [or Ru‐substituted TiN(001) layers] and three atomic layers of hcp Ru(0001) layers, while fixing the bottom‐most TiN layer. To prevent any undesired interactions along the *c*‐direction, all slab models were separated by a vacuum region of at least 15 Å in the *c*‐direction, and a dipole correction was applied. All DFT geometry optimization calculations proceeded until all forces were smaller than 0.01 eV Å^−1^.

The interfacial energy (*E*_inter_) was calculated using,

(1) $$E_{\mathrm{inter}}=E_{\mathrm{total}}-\;E_{\mathrm{Ru}\;\mathrm{slab}}-E_{\mathrm{TiN}\;\mathrm{matrix}}$$

where *E*_total_, *E*_Ru slab_, and *E*_TiN matrix_ are the total energies of the system, the Ru slab, and the TiN matrix with or without the Ru atoms, respectively. The HER Gibbs free energy was determined based on the computational hydrogen electrode (CHE) approach using,

(2) $$\Delta G=\Delta E+\Delta \mathrm{ZPE}+\int C_{p}\mathrm{dT}-T\Delta S$$

where *E*, ZPE*, C_p_, T*, and *S* are the total energies calculated from DFT, the zero‐point energy, the heat capacity, temperature, and entropy, respectively.^[^ ^53^, ^54^ ^]^

## Conflict of Interest

The authors declare no conflict of interest.

## Supporting information

Supporting Information

## Data Availability

The data that support the findings of this study are available from the corresponding author upon reasonable request.
