# Supplementary material for: Ammonolysis‐Driven Exsolution of Ru Nanoparticle Embedded in Conductive Metal Nitride Matrix to Boost Electrocatalyst Activity
Source: Adv Sci (Weinh). 2024 Apr 6;11(24):2309819. doi: 10.1002/advs.202309819 (PMC11200002; doi:10.1002/advs.202309819)
Supplement: Supplementary file 1 — Supporting Information [file ADVS-11-2309819-s001.pdf]

ADVANCED SCIENCE
Open Access

Supporting Information

for *Adv. Sci.*, DOI 10.1002/advs.202309819

Ammonolysis-Driven Exsolution of Ru Nanoparticle Embedded in Conductive Metal Nitride Matrix to Boost Electrocatalyst Activity

So Yeon Yun, Sangseob Lee, Xiaoyan Jin, Aloysius Soon* and Seong-Ju Hwang**

Supporting Information

Ammonolysis-Driven Exsolution of Ru Nanoparticle Embedded in Conductive Metal Nitride Matrix to Boost Electrocatalyst Activity

So Yeon Yun, Sangseob Lee, Xiaoyan Jin, Aloysius Soon,* and Seong-Ju Hwang**

S. Y. Yun, S.-J. Hwang

Department of Materials Science and Engineering, College of Engineering,

Yonsei University, Seoul, 03722, Republic of Korea

E-mail: hwangsju@yonsei.ac.kr (S.-J. H.)

S. Lee, A. Soon

Center for Artificial Synesthesia Materials Discovery, Department of Materials Science and Engineering, Yonsei University, Seoul, 03722, Republic of Korea

E-mail: aloysius.soon@yonsei.ac.kr (A.S.)

X. Jin

Department of Applied Chemistry, University of Seoul, Seoul, 02504, Republic of Korea

E-mail: xjin@uos.ac.kr (X.J.)

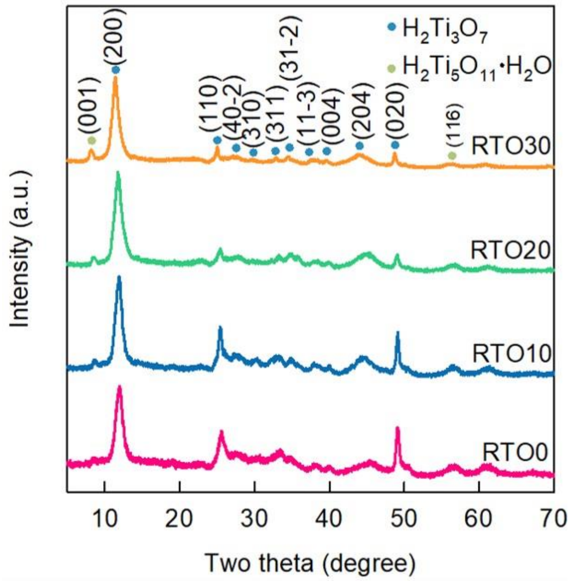

Figure S1. Powder X-ray diffraction (XRD) patterns of the precursor $Ti_{1-x}Ru_xO_2$ nanowires.

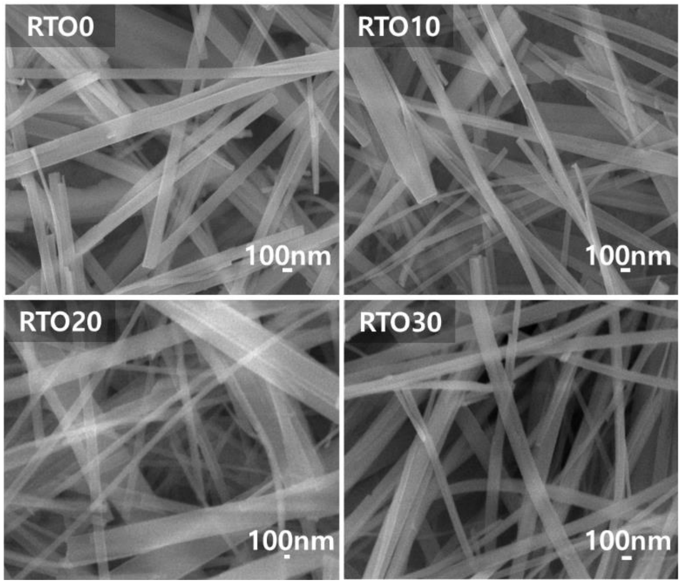

Figure S2. Field emission-scanning electron microscopy **(**FE-SEM) images of the precursor $Ti_{1-x}Ru_xO_2$ nanowires.

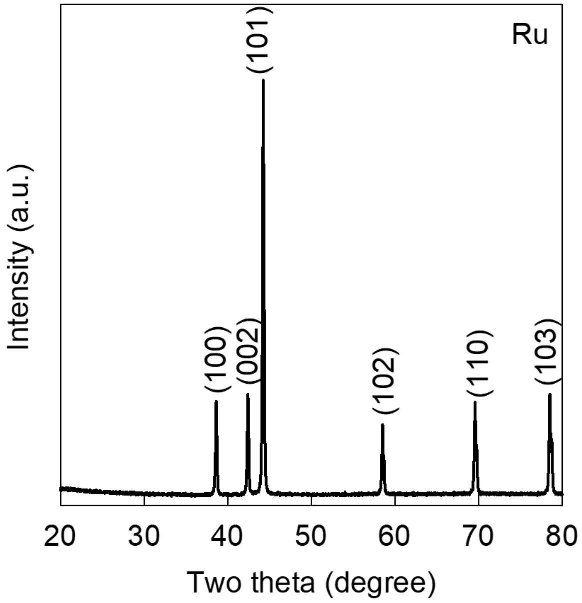

Figure S3. Powder XRD pattern of NH_3-treated RuO_2 material.

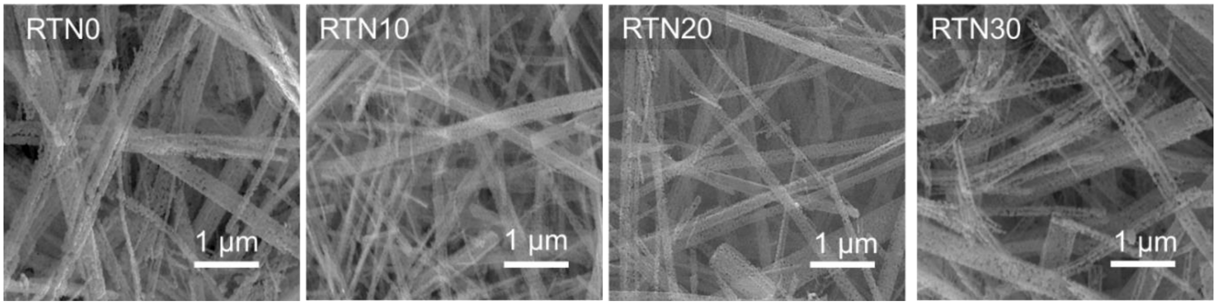

Figure S4. FE-SEM images of holey Ru−TiN nanotubes.

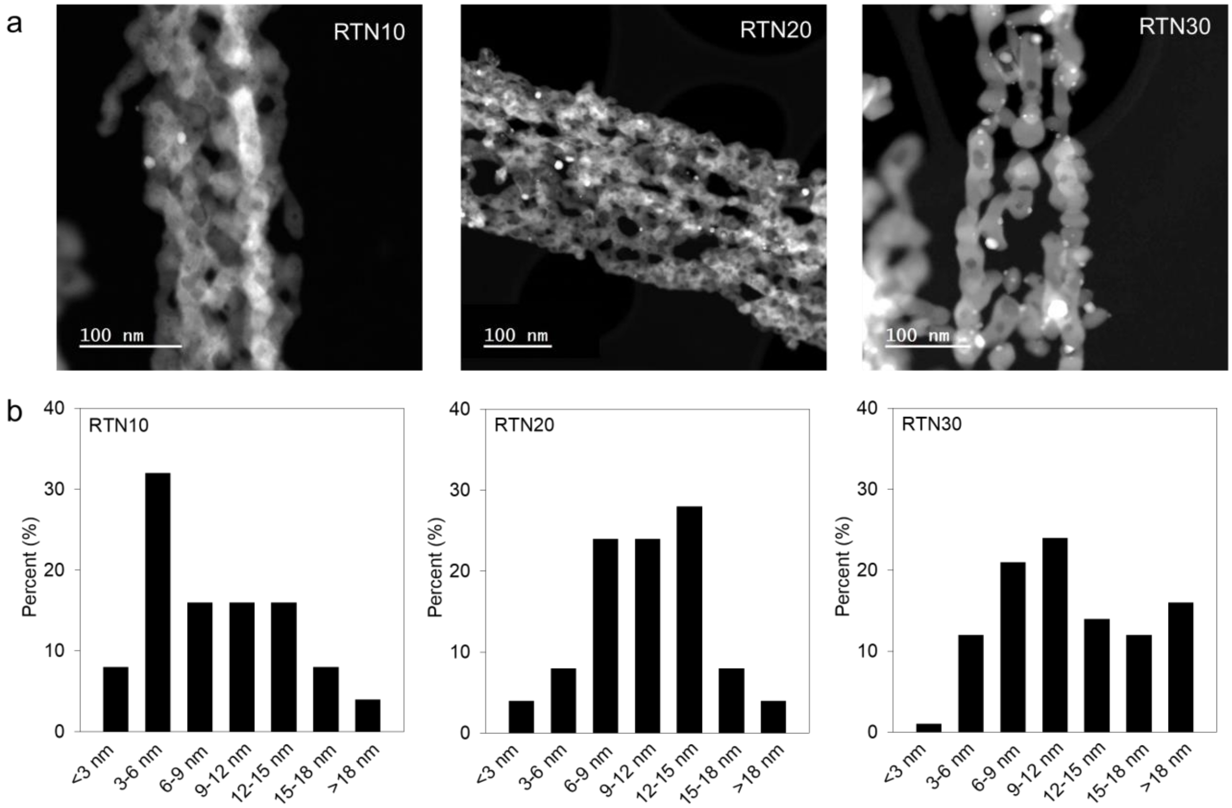

Figure S5. (a) Scanning transmission electron microscopy (STEM) images and (b) the size distribution data of Ru nanoparticles in RTN materials.

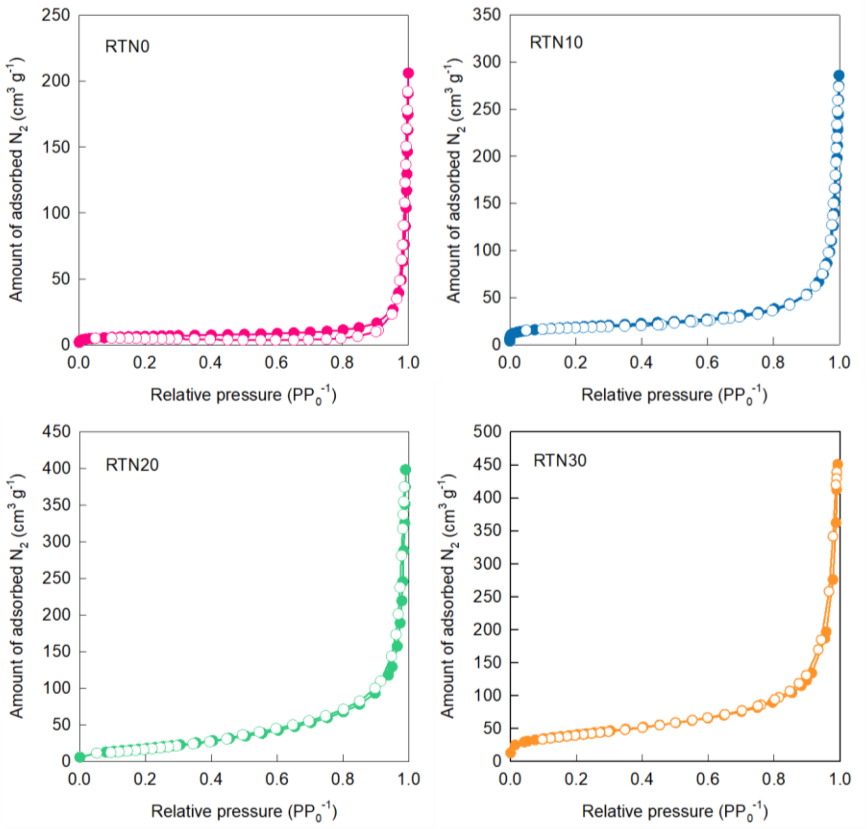

Figure S6. N_2 adsorption–desorption isotherms of Ru–TiN nanotubes.

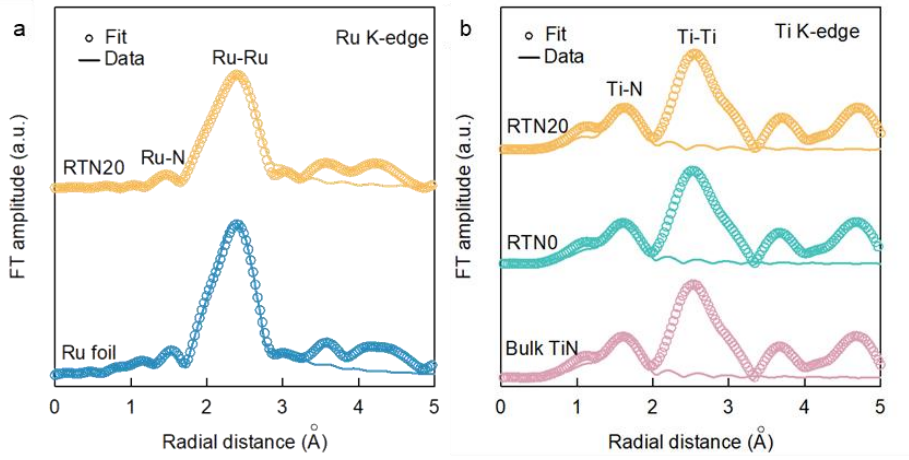

Figure S7. (a) Ru K-edge and (b) Ti K-edge extended X-ray absorption fine structure (EXAFS) fitting data.

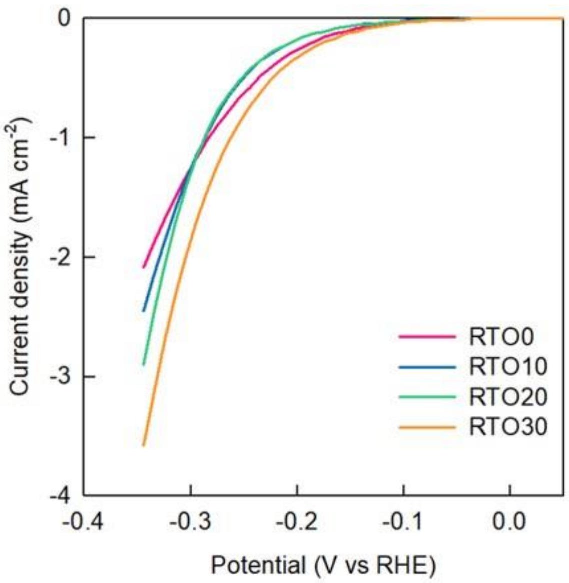

Figure S8. Linear sweep voltammetry (LSV) curves of the precursor $Ti_{1-x}Ru_xO_2$ nanowires.

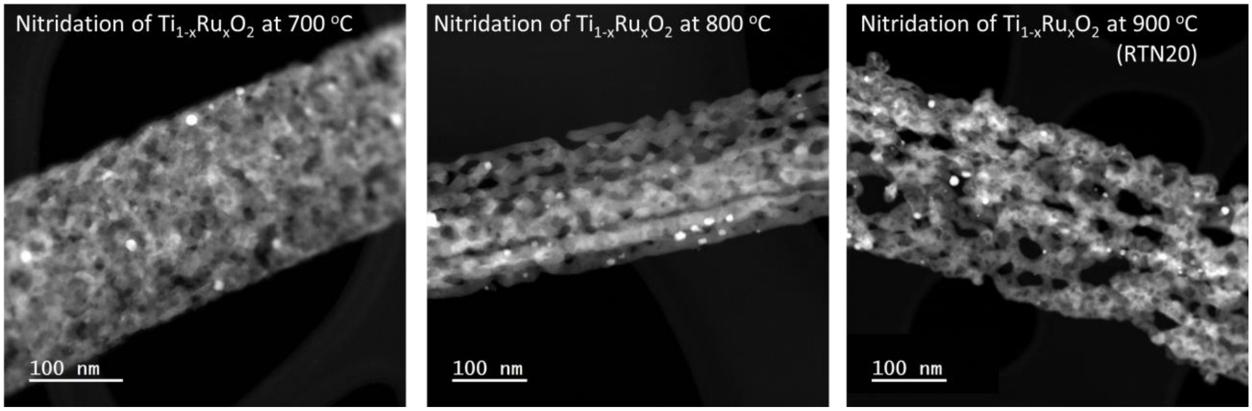

Figure S9. STEM images of RTN20 prepared at different reaction temperatures.

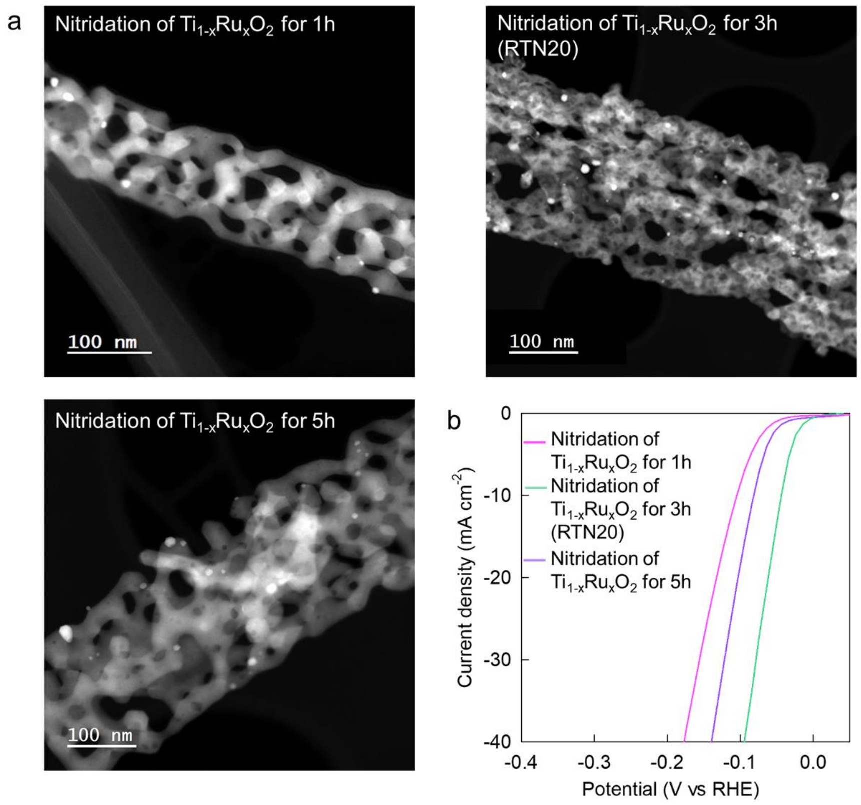

Figure S10. (a) STEM images and (b) LSV curves of RTN20 prepared with different reaction times.

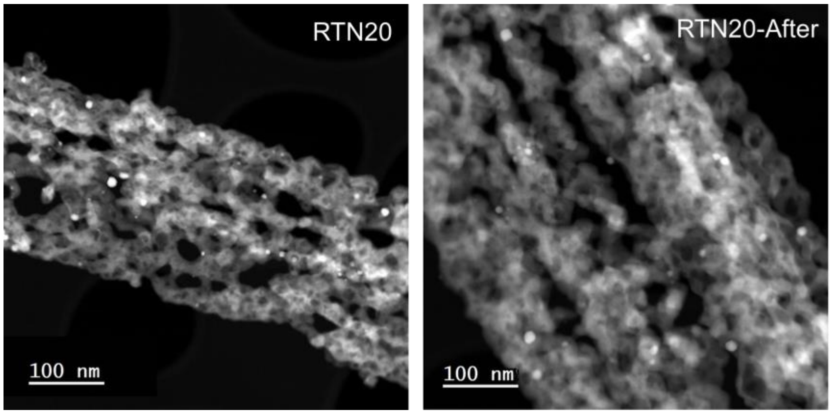

Figure S11. STEM images of RTN20 before and after the long term hydrogen evolution reaction (HER) activity test.

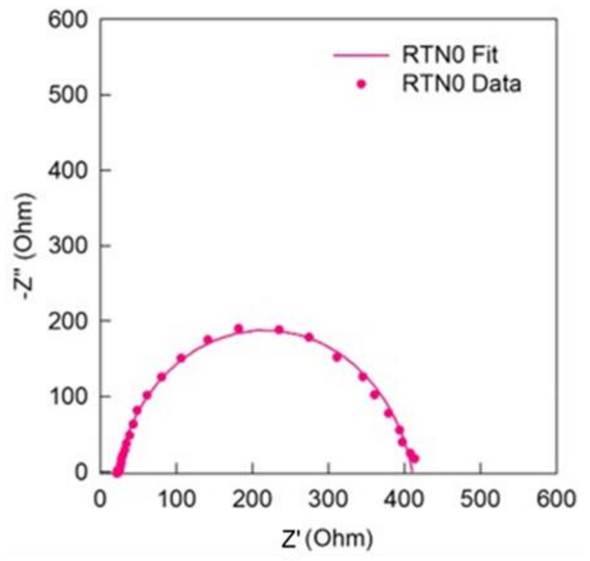

Figure S12. Nyquist plot for Ru-free RTN0.

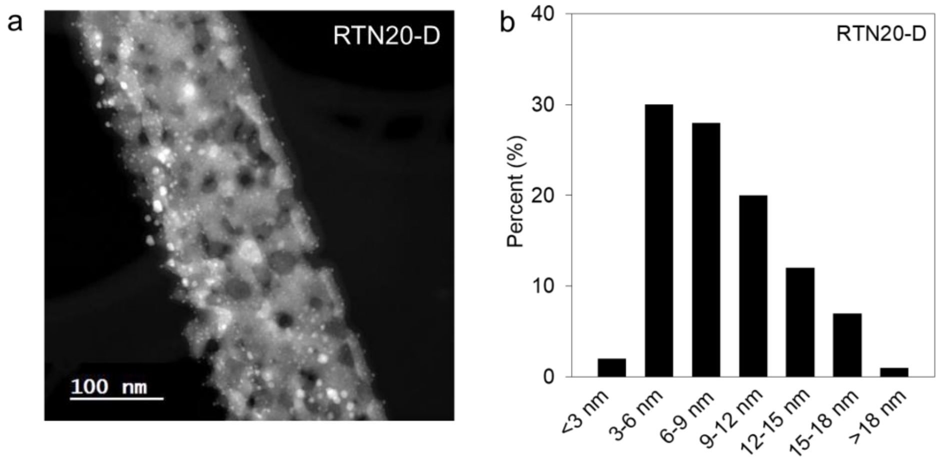

Figure S13. (a) STEM image Ru-deposited RTN20-D and (b) the size distribution data of Ru nanoparticles in Ru-deposited RTN20-D.

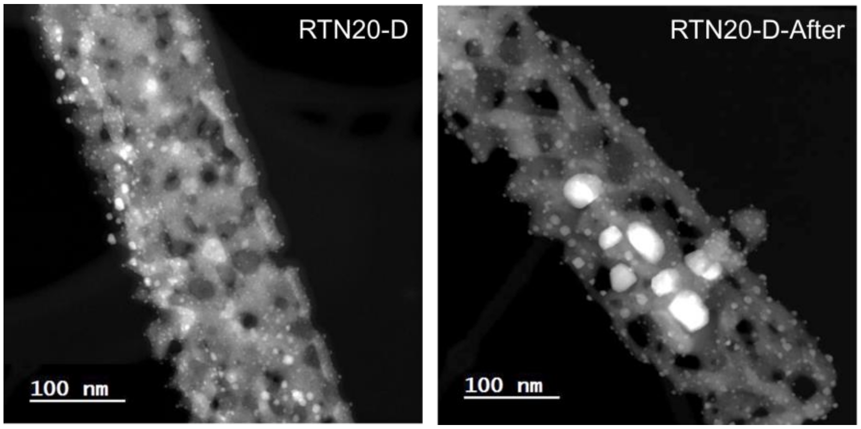

Figure S14. STEM images of RTN20-D (deposition) before and after the long term HER activity test.

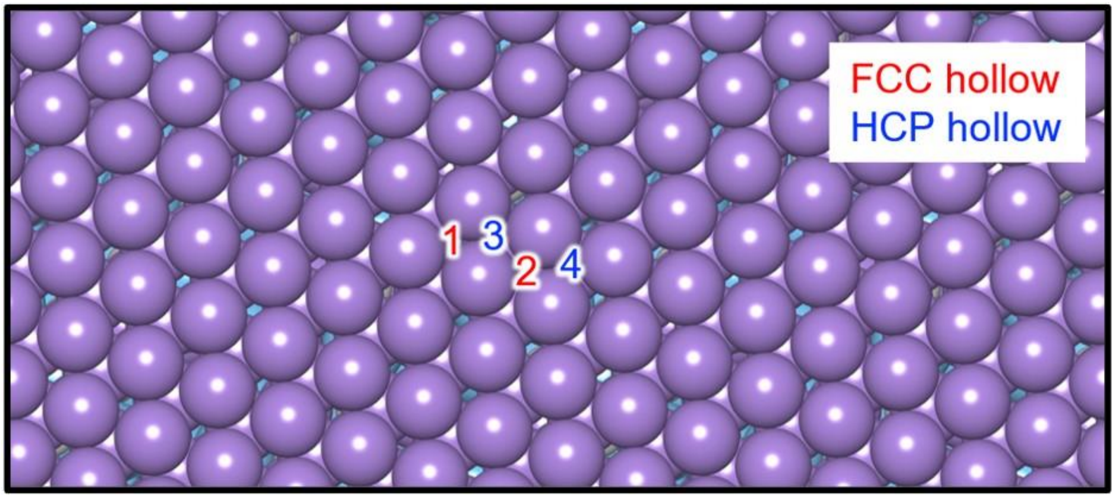

Figure S15. Considered H adsorption sites for both RTN and RTN-D.

Table S1. Lattice parameters of the precursor $Ti_{1-x}Ru_xO_2$ nanowires.

Material	a (Å)	b (Å)	c (Å)	Unit cell volume (Å)
RTO0	16.67	3.69	5.68	349.39
RTO10	16.67	3.69	5.69	350.00
RTO20	16.68	3.69	5.71	351.45
RTO30	16.68	3.69	5.72	352.06

Table S2. Comparison of the electrocatalytic activity with Ru metal-based catalysts toward HER.

Material	Overpotential (10 mA cm^{-2})	ref
RTN20	43	This work
Ru NP/$W_{18}O_{49}$	118	1
Ru@$Ti_3C_2T_x$	46	2
Ru/BN@C	35	3
Ru/HMCs-500	48	4
Ru/RuS_2	45	5
Ru@Co/N-CNT	92	6
Ru-HPC	62	7
Ru@MWCNT	13	8
Ru/Ni_2P	89	9
Ru/NG-750	53	10
Ru-RuO_2/CNT	63	11
Ru/MoS_2/CP	96	12

Table S3. DFT calculated HER Gibbs energy depending on the adsorption site and structure.

Structure	Adsorption site	HER Gibbs energy (ΔG_{H^*})
RTN	1	–0.708
	2	–0.818
	3	–0.640
	4	–0.745
RTN-D	1	–0.742
	2	–0.850
	3	–0.682
	4	–0.777

Table S4. Dissolved metal ion concentration after stability test.

Material	Ru concentration [ppb]
RTN20	No detected
RTN20-D	105.471
Limit of detection	0.006

REFERENCES

[1] Y. Pi, Z. Qui, Y. Sun, H. Ishii, Y.-F. Liao, Z. Zhang, H.-Y. Chen, H. Pang, *Adv. Sci.* **2023**, *10*, 2206096.

[2] Y. Yang, Z. Yu, X. An, X. Duan, M. Chen, J. Zhang, X. Hao, A. Abudula, G. Guan, *Int. J. Hydrogen Energy* **2023**, *48*, 9163.

[3] A. Salah, H.-D. Ren, N. Al-Ansi, H. Tan, F. Yu, L. Yanchun, B. M. Thamer, A. Al-Salihy, L. Zhao, Y. Li, *J. Colloid Interface Sci.* **2023**, *644*, 378.

[4] X. Ma, H. Xiao, Y. Gao, M. Zhao, L. Zhang, J. Zhang, J. Jia, H. Wu, *J. Mater. Chem. A* **2023**, *11*, 3524.

[5] J. Zhu, Y. Guo, F. Liu, H. Xu, L. Gong, W. Shi, D. Chen, P. Wang, Y. Yang, C. Zhang, J. Wu, J. Luo, S. Mu, *Angew. Chem. Int. Ed.* **2021**, *60*, 12328.

[6] Z. Liu, X. Yang, G. Hu, L. Feng, *ACS Sustain. Chem. Eng.* **2020**, *8*, 9136.

[7] T. Qiu, Z. Liang, W. Guo, S. Gao, C. Qu, H. Tabassum, H. Zhang, B. Zhu, R. Zou, Y. Shao-Horn, *Nano Energy* **2019**, *58*, 1.

[8] D. H. Kweon, M. S. Okyay, S.-J. Kim, J.-P. Jeon, H.-J. Noh, N. Park, J. Mahmood, J.-B. Baek, *Nat. Commun.* **2020**, *11*, 1278.

[9] J.-Q. Chi, X.-Y. Zhang, X. Ma, B. Dong, J.-Q. Zhang, B.-Y. Guo, M. Yang, L. Wang, Y.-M. Chai, C. Liu, *ACS Sustain. Chem. Eng.* **2019**, *7*, 17714.

[10] R. Ye, Y. Liu, Z. Peng, T. Wang, A. S. Jalilov, B. I. Yakobson, S.-H. Wei, J. M. Tour, *ACS Appl. Mater. Interfaces* **2017**, *9*, 3785.

[11] M. Zhang, J. Chen, H. Li, P. Cai, Y. Li, Z. Wen, *Nano Energy* **2019**, *61*, 576.

[12] J. Liu, Y. Zheng, D. Zhu, A. Vasileff, T. Ling, S.-Z. Qiao, *Nanoscale* **2017**, *9*, 16616.
